# Metformin hydrochloride entrapment in sorbitan monostearate for intestinal permeability enhancement and pharmacodynamics

**DOI:** 10.1038/s41598-021-99649-3

**Published:** 2021-10-11

**Authors:** Omar Y. Mady, Adam A. Al-Shoubki, Ahmed A. Donia, Waseem Qasim

**Affiliations:** 1 Pharmaceutical Technology Department, Faculty of Pharmacy, Tanta University, Tanta, Egypt; 2 Pharmaceutics and Industrial Pharmacy Department, Faculty of Pharmacy, Omar Al-Mukhtar University, Al-Bayda, Libya; 3 Department of Pharmaceutical Technology, Faculty of Pharmacy, Menoufia University, Shebeen El-Kom, Egypt; 4 Department of Laboratory Sciences and Pharmaceutics, Faculty of Pharmacy, Ahl Al Bayt University, Karbala, Iraq

**Keywords:** Biotechnology, Drug discovery

## Abstract

Penetration enhancement of metformin hydrochloride via its molecular dispersion in sorbitan monostearate microparticles is reported. This represents basic philosophy to maximize its entrapment for maximum penetration effect. Drug dispersion in sorbitan monostearate with different theoretical drug contents (TDC) were prepared. Products showed excellent micromeritics and actual drug content (ADC) increased by increasing TDC. The partition coefficient of the drug products showed huge improvement. This indicates the drug entrapped in the polar part of sorbitan monostearate as a special image which effects on the drug release. The drug permeation profiles from the different products are overlapped with nearly equal permeation parameters. The permeation results suggested the main driving force for improving the drug paracellular pathway is its dispersion in sorbitan monostearate and is independent of ADC. Pharmacodynamic of the products showed a significant improvement than the drug alone at p ˂ 0.05. ANOVA test indicated the insignificant pharmacodynamic difference between the low, middle, and high ADC of the products. An excellent correlation founded between the drug permeation and pharmacodynamic precents. Drug permeation driving force via the paracellular pathway is its entrapment in sorbitan monostearate and independent on ADC. The technique is simple and the products had excellent micromeritics.

## Introduction

Metformin Hydrochloride is one of the worldwide drugs used for the treatment of diabetic II patients. The drug is considered safe and does not produce a hypoglycemic effect^1^. It has some drawbacks concerned with gastro-intestinal disorders especially with an older patient^2^. The marketed doses of the drug are 3 doses, 500, 850, and 1000 mg per tablet with low bioavailability^3^. The biopharmaceutical classification system classifies metformin hydrochloride as a class III drug^4^. This group of drugs has high solubility but low intestinal permeability^4^. Then, it can understand the low bioavailability of the drug and consequently the high dose used in addition to the abdominal side effect.

Metformin hydrochloride was found to be absorbed (about 90%) via the Paracellular pathway^5,6^. The high solubility of the drug and its Paracellular pathway absorption suggested a rapid and complete drug absorption. But the absorption of metformin hydrochloride is limited. It was reported that the addition of tween 80 to the drug led to an increase in all drug permeation parameters^7,8^. That is due to the role of non-ionic surfactants on enhancing metformin absorption via the paracellular pathway^7,8^. There is a significant saturable component of the paracellular pathway that limited the absorption of the highly water-soluble drug. The presence of tween 80 reduces the saturable paracellular pathway by electrostatic interactions between. the opposite charges of diffused substances (drug and tween 80) and the anionic residues of the lateral space and or tight junctions^5,6,9,10^. A novel “sponge” hypothesis was formulated to explain how metformin could be absorbed across human intestinal through the predominantly paracellular mechanism^5,10^.

Mady et al.^7^ succeed to prepare sorbitan monostearate as microparticles containing metformin in the molecular state by using the rapid congealing technique. The molecular state of the drug in sorbitan monostearate was proved by different instrumental analyses. The authors suggested and used a modified non-everted sac technique in the discussion of the in-vitro drug permeation mechanism. They found that the addition of tween enhanced the drug permeation from the rabbit intestinal sac. The drug permeation enhancement was more pronounced from sorbitan monostearate entrapped drugs. The permeation parameters (permeation coefficient, total penetration percent and drug absorption enhancement %) are markedly increased from sorbitan monostearate microparticles encapsulating drug. The authors concluded that, as a result of the drug polarity, the drug is encapsulated in the polar part of the surfactant which led to a huge increasing the encapsulated drug partition coefficient. Emulsification of sorbitan monostearate microparticles encapsulated drug in phosphate buffer pH 6.8 by using tween 80 indicated the change of the surface again to hydrophilic. This image may be responsible for the above permeation parameters as a result of the correction of the HLP value (hydrophilic-lipophilic balances) of the image, which could be easily diffused through the paracellular pathway^7^.

Accordingly, the aim of this work is trying to investigate the penetration driving force of the drug via the paracellular pathway based on the previously reported by the author that, the drug dispersion in sorbitan monostearate has higher paracellular pathway penetration^7^. Then studying the relationship between increasing the actual drug content on the penetration effect to achieve the maximum drug dispersed in sorbitan monostearate for maximum penetration effect and consequently maximum pharmacodynamic effect. The pharmacodynamic study would be tested in diabetic rats. The micromeritics properties of the prepared products should be also studied, which are essential in the manufacture processing. At the end trying to find a correlation between the two normally related parameters for a drug, drug permeation enhancement percent and the drug pharmacodynamic enhancement percent.

## Materials and methods

### Materials

Metformin hydrochloride (HCl), was purchased from El-Nasr Pharmaceutical Chemical Co (Egypt), Sorbitan monostearate of research-grade was purchased from Altas Chemie, IC GmbH (Germany). All other chemicals were of analytical grade and used as received.

### Methods

#### Dispersion of metformin HCl in sorbitan monostearate

Metformin HCl is dispersed in sorbitan monostearate by using the melting method. The required amounts of metformin HCl and sorbitan monostearate to prepare the solid dispersions of 25%, 33.33%, 50%, 66.66% and 75% theoretical drug content (TDC) in sorbitan monostearate were weight and physically mixed^11^. The prepared physical mixtures were melted at 60 °C ± 5 while stirring until clear molten solutions were obtained to assure from molecular dispersion of the drug in the carrier. The molten solutions were slowly cooled at room temperature while stirring to form sold masses. The products obtained were grinding, sieving, and stored at room temperature. The product particles, which passed from sieve size 600 µm was used for further studies^7,11^.

#### Characterizations of the prepared drug-sorbitan monostearate solid dispersions

##### Determination of the actual drug content % [ADC]

An accurate amount of each solid dispersion product containing 20 mg as TDC was weighed and then dissolved in 100 ml of 0.1 N HCl at 60 °C. The solution may be filtered if necessary and measure spectrophotometry at 232 λ max using 0.1 HCl as a blank. Sometimes dilutions may be carried out and the procedure was repeated in triplicate. The mean actual drug content and encapsulation % were calculated using Eqs. (1) and (2)^11,12^.

1 $$\mathrm{Theoretical\, drug \,content }\;(\mathrm{TDC}) =\frac{\mathrm{drug \,total}}{(\mathrm{drug \,total}+\mathrm{sorbitan \,monostearate})}\times 100$$

2 $$\mathrm{Actual \,drug \,content }\;(\mathrm{ADC}) =\frac{\mathrm{Actual \,drug \,content \,total}}{(\mathrm{drug \,total}+\mathrm{sorbitan \,monostearate})}\times 100$$

##### Flow properties

The angle of repose method was used to study the follow property of the sorbitan monostearate-metformin solid dispersion products. The measuring was done by maintaining the funnel at a fixed height from a smooth glass surface in all experiments. The samples were passed through a funnel to form a stable cone^13,14^. The angle of repose (ɵ) was calculated by using Eq. (3).

3 $$ \theta = {\text{tan}}\;\left( {{\text{h}}/{\text{r}}} \right) $$

where θ = angle of repose, h = height of cone, r = radius of the cone base^13,14^.

##### Measurements of densities

A fixed weight of sorbitan monostearate-metformin solid dispersion product was carefully introduced into a 50 ml graduated cylinder. The bulk volume (V0) was measured in cm^3^. Then dropping the cylinder from a high of 2.5 cm at one-second intervals. The procedure was repeated till no further change in volume was noted. The tapped volume (Vt) was measured cm^3^^15–17^. The densities parameters of the granules were calculated according to Eq. (4)^18^, Eq. (5)^18^, and Eq. (6)^19^.

4 $$\mathrm{Bulk \,density }=\frac{\mathrm{weight \,of \,the \,sample \,in \,gm}}{\mathrm{volume \,in \,cm}^{3} (\mathrm{V}0)}$$

5 $$\mathrm{Tapped \,density }=\frac{\mathrm{weight \,of \,the \,sample \,in gm}}{\mathrm{volume \,in \,cm}^{3} (\mathrm{Vt})}$$

6 $$\mathrm{Carr's\, index}= \frac{\mathrm{Tapped \,density}-\mathrm{ Bulk \,density}}{\mathrm{Tapped \,density}}\times 100$$

##### Experimentally determination of partition coefficients (Log P) by using n-octanol–water

A 20 mg of either pure drug or that of each product containing 20 mg metformin HCl determined from the actual drug content was taken and dissolved in 20 ml of n-octanol. Then 20 ml of distilled water was added to the previous n-octanol solution while stirring. The formed system was transferred into a separating funnel and allowed to equilibrate. The concentration of the drug diffused from the organic phase (n-octanol) to the aqueous was measured spectrophotometrically at 232 λ max. using Eq. (7)^7,20^

7 $$\mathrm{Log \,p }=\mathrm{log}\left[ \frac{solute \,unionized \,in \,octanol}{solute \,ionized \,in \,water}\right]$$

##### Drug release profile

To the USP paddle dissolution apparatus, an accurate weight of the product containing 500 mg of Metformin HCl [calculated according to the determined actual drug content] was added^21^. The release solution was 900 ml of 0.1 N HCl with a maintained temperature at 37 ± 0.5 °C. The stirring rate was 100 rpm. The samples of 5 ml were taken at predetermined time intervals for determining the cumulative drug release. A new release medium was added to replenish each sample withdrawn. Sometimes dilutions may be carried out and the resulting solutions were measured at 232 λ max using 0.1 HCl as a blank. The procedure is carried out in triplicate^7^.

##### Non-everted sac model as a tool to evaluate the intestinal permeability

The method steps employed to evaluate the drug permeability profile from the modified non-everted sac method are modified experimental procedures described by references^22–25^.

- Preparation of non-everted intestinal sacs:
  The animal used in this study was a Male albino rabbit with a weight of 2 kg obtained from the Tanta animal house. All procedures were approved and regularly controlled by the Animal Ethics Committee of Faculty of Pharmacy Tanta University (No: 2212018) and all experiments were performed by the guidelines and regulations of this committee. All the procedures were also carried out in full accordance with the ARRIVE guidelines 2020^26^, and adequate care was taken to minimize pain and discomfort for animals. Upon confirmation of loss of the pain reflex, the animal was sacrificed. A midline longitudinal incision of 3–4 cm was made, and the small intestine was located. A 14 cm segment of the intestine was used to prepare the sac. The lumen of the intestinal segment was washed with buffer to remove any solid material. Using a surgical thread, a side of the segment of the small intestine could be tied. The fresh intestinal sac was then filled with buffer, tied to the other side with surgical thread, and checked for leaks. The prepared segments after each step were placed in continuous aerated phosphate buffer and used for studying the drug permeation after filling with the perfusion solution^7^.
  An amount of Sorbitan monostearate product containing 50 mg of Metformin HCl as an actual drug content was accurately weighed and dispersed in 4 ml of pH 6.8 phosphate buffer. One ml of tween 80 added. The fresh intestinal sac segment was then emptied from the buffer solution. The intestinal sac was then filled with prepared perfusion solution, tied with surgical thread, and tested for leaks. The segment length and diameter were measured for surface area determination^7^.
- Drug permeation profile study
  The prepared segment was suspended on the shaft of the USP dissolution apparatus. The outside of the sac medium (permeation medium) was 900 ml of phosphate buffer (pH 6.8) with continuous aeration and maintained the temperature at 37 ± 0.5 °C. The stirring rate was 50 rpm. Samples of 5 ml were taken at predetermined time intervals and the new release medium was added to replenish each sample taken. The amount of drug permeated from the segment to the medium was determined spectrophotometrically at 232 λ max^7^.
- Determination of permeability coefficient
  The use of Fick`s law for the determination of the permeability coefficient (apparent permeability) of the drug across the isolated rat intestine was previously reported^7,27,28^. A simplified equation could be written as Eq. (8).

8 $$ {\text{dM}}/{\text{dt}} = {\text{PSCd}} $$

The variables M and Cd could be determined by analysis of mucosal fluid. The surface area [S] could be calculated by considering the intestinal sac a cylinder. Then, M/SCd could be calculated and plotted against time. The slope of the linear part of the plot is the permeability coefficient (P), which has the units of velocity (cm/s). The slope of the linear part of the curve was determined by linear regression^27–30^.

##### Pharmacodynamics study

- Animals
  The animal used in this study was the male albino Wister rats aged 7–8 weeks with a weight of 150–200 g. All procedures were approved and regularly controlled by the Animal Ethics Committee of Faculty of Pharmacy Tanta University (No: 2212018) and all experiments were performed by the guidelines and regulations of this committee. All the procedures were also carried out in full accordance with the ARRIVE guidelines 2020^26^, and adequate care was taken to minimize pain and discomfort for animals. The housing of the animal was at an ambient temperature of 25 ± 1 °C and relative humidity of 45–55% with a 12 h each of dark and light cycles. They fed was pellet diet and water ad libitum.
- Induction of the experimental diabetes
  After overnight fasting of the animals, diabetes was induced by a single intraperitoneal injection of a freshly prepared solution of streptozotocin (50 mg/kg body weight) in 0.1 M citrate buffer (pH 4.5)^31,32^. The animals were allowed to drink 5% glucose solution as a solution for the hypoglycaemic effect of the drug^33^. On the third day of streptozotocin injection, the rats fasted for 6 h and blood was withdrawn from the tail vein. The blood glucose level was measured by using Accu-Chek active (Accu-chek active test strip). Rats that had fasting blood glucose levels > 250 mg/dl were considered to be diabetic and were used to monitor the efficacy of metformin formulations^34^.
- Determination of the hypoglycaemic effect of metformin HCl
  For only 15 min on the day of the experiment, the rats were given free access to the pellet. To provide a stable blood glucose level, the food was restricted but the rats were given free access to water for 2 h. Then, the formulations were dispersed in water at a concertation of 30 mg/ml, and 0.125 ml tween 80 was added for each ml. From the prepared tested dispersion, 1 ml was administered orally to each rat. At time intervals of (0, 0.25, 0.5, 1, 2, 3, 4, 5, 6, 7, and 8) hours, blood samples were withdrawn from the tail vein. The blood glucose was measured by using Accu-Chek active (Accu-chek active test strip). The blood glucose level was plotted as a function of time. The area above the curve was determined and used for monitoring the efficacy of different formulations. The amount of reduction in blood glucose level was also calculated and plotted as a function of time^7,35^.
- Statistical analysis
  Statistical analysis of the in-vivo data was carried out by applying the ANOVA test. The data were analyzed by one-way analyses of variance (ANOVA) with blood glucose level as an independent factor.

### Ethics approval

All procedures of animal study were approved and regularly controlled by the Animal Ethics Committee of Faculty of Pharmacy Tanta University (No: 2212018) and all experiments were performed in accordance with the guidelines and regulations of this committee. All the procedures were also carried out in full accordance with the ARRIVE guidelines 2020^26^.

## Results and discussion

The work aims to determine the drug permeation driving force through the Paracellular pathway reported by the authors from sorbitan monostearate microparticles encapsulated the drug^7^. In that work, it was proved the molecular dispersion of the drug led to improving its Paracellular pathway permeation. To be assured from complete dispersion of the drug molecules and sorbitan monostearate, the solid dispersion is prepared by melting the physical mixture of the required amounts of the drug and the carrier. In addition, it could be expected there is no loss in both components of the solid dispersion as a result of the absence of a third component, which can dissolve either of the components or both. This led to assurance from nearly remaining the ratios of the components.

The effect of drug dispersion in the wax matrix on the drug processing could be noticed from the Table 1. The flow property of all drug-matrices ratios was markedly improved from fair to passable flow in the case of the pure drug to very free-flowing except that prepared with 75% TDC, which is free-flowing. That may be due to the low concentration of the wax matrix compared to other ratios. The closest of the angle of repose values on using different concentrations of the wax matrix may be due to the method of preparation, which is based on melting the drug and the matrix to form a clear melted solution before solid mass formation after cooling. Therefore, the homogeneity of the dispersion of the drug in the dispersed substance could be expected. These results indicate the advantage of using sorbitan monostearate as a dispersed matrix since it improves the flowability of the dispersed drug which is sometimes considered as a limiting step in the pharmaceutical manufacture processes. In addition, to assure the homogeneity of using different concentrations of the dispersed media, it will be preferring to use the melting method to get a clear melt solution. This may be a disadvantage in using the melting technique for thermos-labile substances but it may be not considered because the melting point of sorbitan monostearate is around 56 °C. The compressibility index of the solid dispersed drug in sorbitan monostearate by using melting method is completely improved from poor compressible substance in case of the pure drug to excellent compressibility substance for all solid dispersed drug-sorbitan monostearate ratios^36^.

**Table 1:** Micromeritic properties of the prepared products.

TDC %	Compressibility %	± SD	Angle of repose	± SD
25	7.907	0.104	28.60	0.530
33	6.009	0.165	29.14	0.082
50	6.100	1.246	29.17	0.089
66	7.872	1.254	29.85	0.681
75	8.411	1.120	31.35	0.037
100	26.767	0.910	39.71	0.550

Since metformin-sorbitan monostearate solid dispersions were prepared by using melting technique, this led to supposes that there is no loss for both drug and matrix and the actual drug content should be equal to theoretical drug content. On the determination of the actual drug content, from a Table 2, it can be noticed that the actual drug content (ADC) is nearly equal to the theoretical drug content (TDC). The deviation of ACD from TDC is nearly constant and is maximum on using 25% TDC. That is maybe due to the insolubility of sorbitan monostearate in the extraction solution. ADC increased nearly parallel to increasing the TDC.

**Table 2:** The actual (ADC) and theoretical (ATC) drug content of the prepared products.

TDC %	ADC %	± SD
25	21.909	0.90
33	31.455	1.29
50	48.727	2.31
66	64.909	1.03
75	73.545	1.16

### Partition coefficient

**Figure 1:**
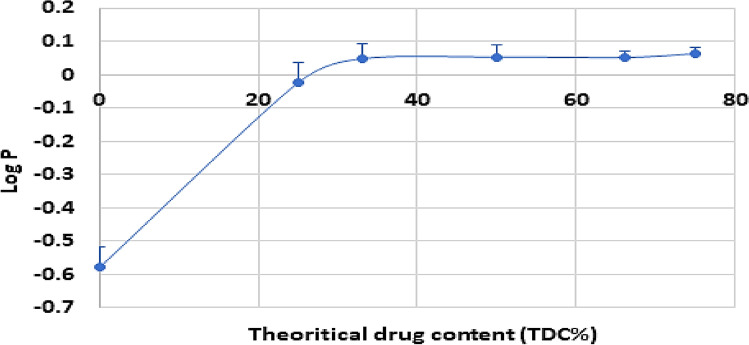
Effect of the theoretical drug content on its partition coefficient.

The partition coefficient of the pure drug and all drug solid dispersion products with different ratios were studied. Figure 1 showed that the partition coefficient of 25% drug dispersed in sorbitan monostearate as solid dispersion increased markedly than that of the pure drug. On using 33% drug in the solid dispersion form, the value of **P**o/w is again increased and remains constant on increasing the drug percentage in the solid dispersion products. Since the drug-sorbitan monostearate solid dispersion is prepared by the melting method to produce a melt clear solution and the partition coefficient value depends on the lipophilicity of the drug, it can be concluded that the melting of the drug led to its dispersion in the polar part of the carrier. In this case, the polar part of the carrier could be only the polar part of an image of the surfactant which is, maybe, the surfactant micelle. This conclusion was also previously reported by the author^7^.

### Dissolution profile

**Figure 2:**
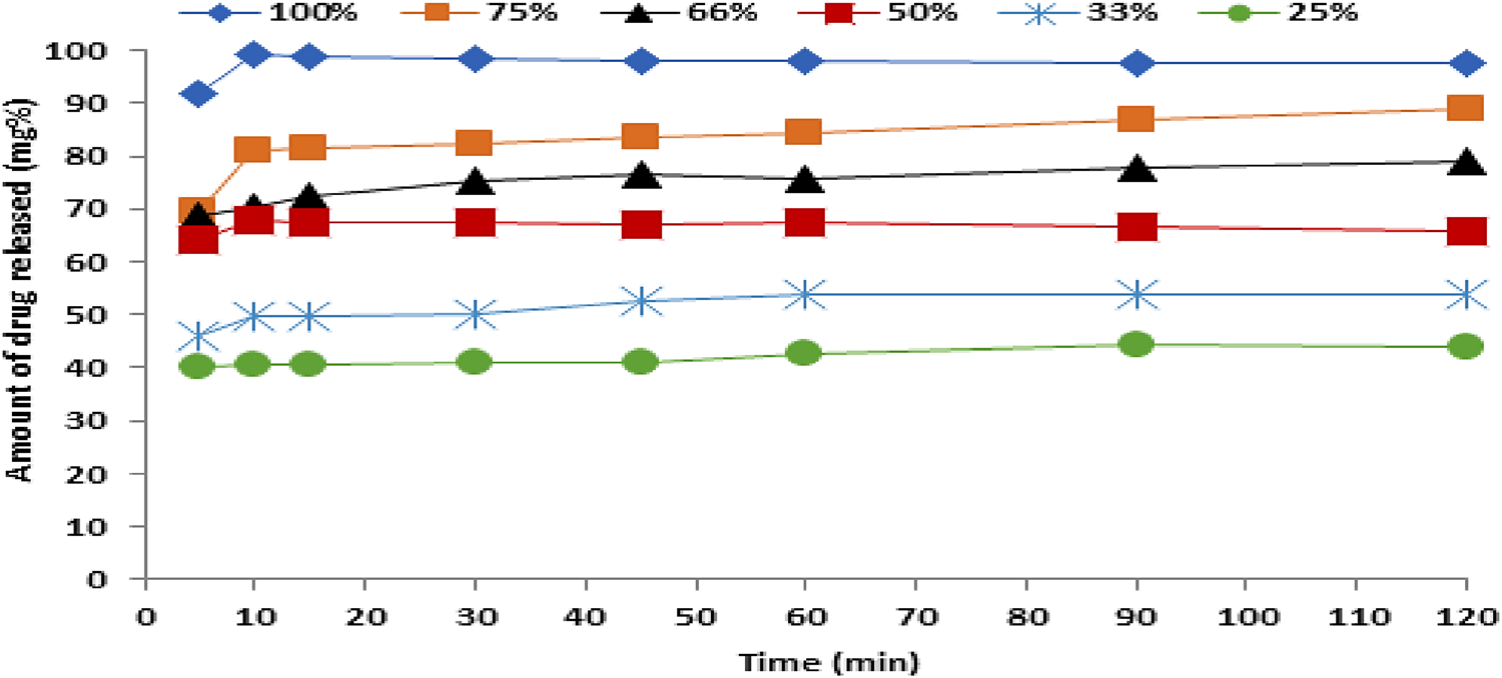
Drug release profile from different prepared drug-span matrices.

The drug release profile from different matrices prepared with different metformin-span ratios was studied. From Fig. 2, it can be noticed that there is rapid initial and incomplete drug release and both depend on the drug-matrix ratio. These two features are well-known and characterized for each drug dispersed in the molecular state in an insoluble matrix which is, maybe, also our case. Increasing the wax ratio led to decreasing the burst effect and total drug release and the reverse could be noticed concerning the drug. That is maybe due to the insolubility of sorbitan monostearate in the dissolution media. In addition, the high solubility of the drug in the dissolution media may enhance the burst effect.

### Permeation profile

The intestinal sacs for assaying the permeability are a quick and sensitive technique for determining the overall intestinal integrity or comparative transport of a specific molecule, with the added benefit of intestinal site-specificity. The apparent permeability [Papp] or permeation coefficient of a molecule through the intestinal barrier could be calculated^37,38^. The benefits of the application of the intestinal sac would be increased after solving the critical points facing its application by the author. Mady et al.^7^ succeed to solve the critical points of the technique by suspension the sac to the dissolution shaft in the dissolution media of the dissolution apparatus. This modification led to creating instead of drug release profile to drug permeation profile on using standard dissolution apparatus. The author discussed the critical points about the suggested solution, which may increase the value of the application of the technique as a modified non-everted sac.

**Figure 3:**
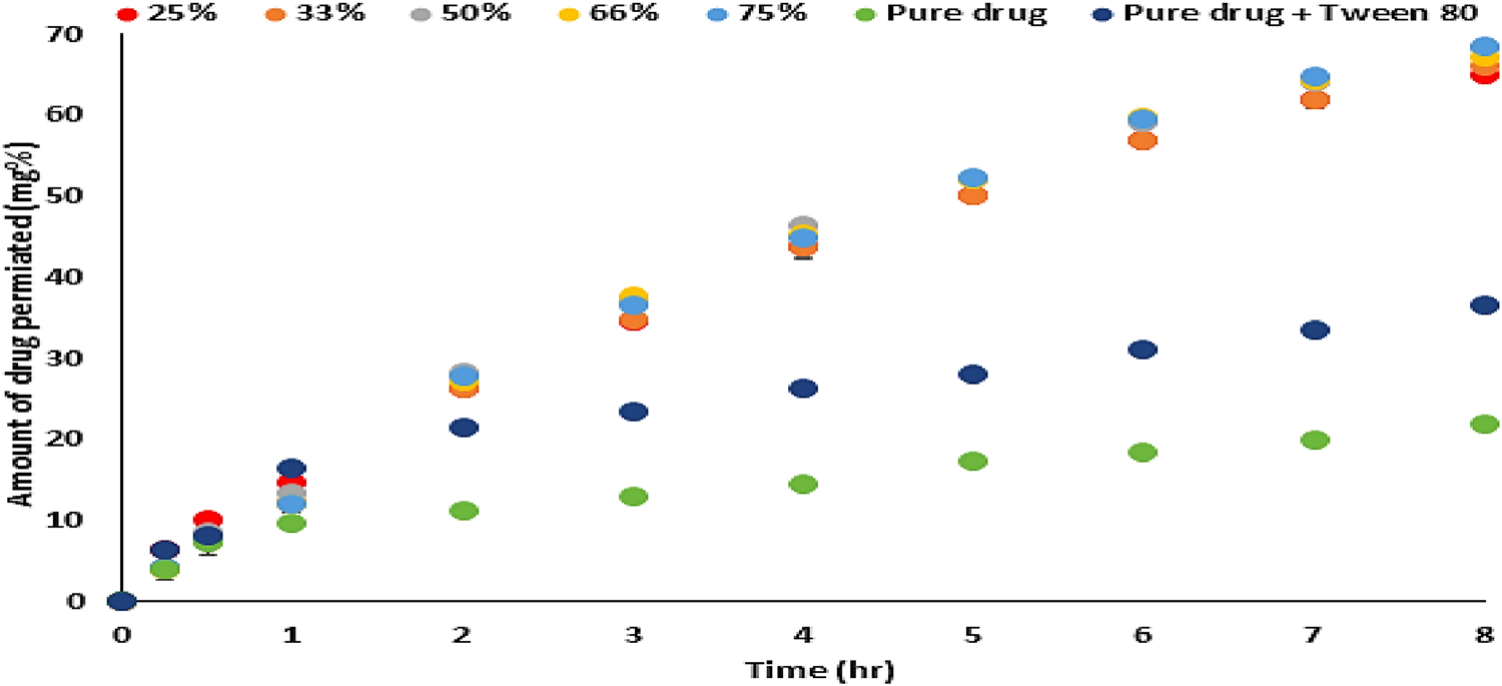
The permeability profiles of the pure drug, drug-tween, and different molecular solid dispersions of the drug in sorbitan monostearate matrices.

Figure 3 showed that the addition of tween 80 to the pure drug enhances its permeability concerning the initial, rate and total drug permeated in the experimental time. That is due to different reported mechanisms^5,7,9,10^ These effects would be more pronounced from the different solid dispersion products of the drug in sorbitan monostearate. The drug permeation profiles from the different drug-sorbitan monostearate solid dispersion products showed an overlap profile style concerning the initial, rate, and the total amount of drug released. This may be due to the drug dispersion in the carrier matrix and the use of an amount of the products containing the same actual drug concentration. Mady et al.^7^ discussed the essential of metformin HCL encapsulation in the molecular state in the carrier to improve its permeability. The conclusion of the author about the essential molecular dispersion of the drug in sorbitan monostearate as a carrier represents the basic philosophy to maximize its entrapment in the matrix. Increasing the initial drug permeated and total drug permeated leads to expecting improving the onset of action and decreasing the drug reported dose. Although metformin is reported to be safe from producing a hypoglycemic effect, decreasing the dose leads to decreasing the GIT side effect especially in older patients.

The permeability parameters of metformin HCl, metformin-tween, and different dispersion of metformin-sorbitan monostearate products across the non-everted sac were calculated and summarized in Table 3. The values of r^2^, in each case, are high enough to consider a good fitting of the calculated permeation data. In every case, there is no lag time. In the case of metformin HCl, the absences of lag time may be due to its high-water solubility which may be responsible for the presence of intercept values with y abscissa in concentration. The presence of an intercept value represents, in this case, the rapid saturation of the Paracellular pathway tissues of the intestinal wall with the drug before drug transport^7^. This finding is reinforced by the fact that about 90% of metformin HCl is absorbed via the Paracellular pathway^5,6^, and the absence of lag time. The addition of tween 80 to the drug led to increasing of all permeation parameters by different reported mechanisms^5,7,9,10^. The permeation parameters of the drug from its different molecular dispersion products in sorbitan monostearate are markedly increased than that from drug-tween although the intercept values are nearly the same. In addition, the permeation parameters of the drug from its solid dispersion products prepared with different ratios are nearly equal which is reflected in the drug absorption enhancement (DAE %). The drug absorption enhancement percent was calculated according to Eq. (9)^7^

**Table 3:** Metformin HCl transferred data through non-everted intestinal sac of pure drug, drug- tween, and different solid dispersions of the drug in sorbitan monostearate.

	r^2^	Papp (cm/s) 10^–4^	Intercept	Total permeation %	DAE %
Pure drug	0.995	4.723	7.631	21.860 (± 0.222)	100.000
Drug-tween	0.997	6.706	15.957	36.524 (± 0.259)	167.0814
25% span	0.985	17.368	16.161	68.387 (± 0.649)	312.8408
33% span	0.973	17.644	16.975	67.032 (± 0.432)	306.6423
50% span	0.982	17.656	17.412	68.159 (± 0.693)	311.7978
66% span	0.984	17.764	15.205	65.972 (± 1.061)	301.7932
75% Span	0.979	18.239	15.586	64.960 (± 0.400)	297.1638

9 $$\mathrm{\% DAE}=\frac{\mathrm{The\, cumulative \,amount \,of \,drug \,penetrated \,from \,the \,dosage \,form}}{\mathrm{The \,cumulative \,amount \,of \,pure \,drug \,penetrated}} \times 100$$

From Table 3, it can be noticed that the drug absorption enhancing percent from different molecular drug solid dispersion percent are nearly equal.

**Figure 4:**
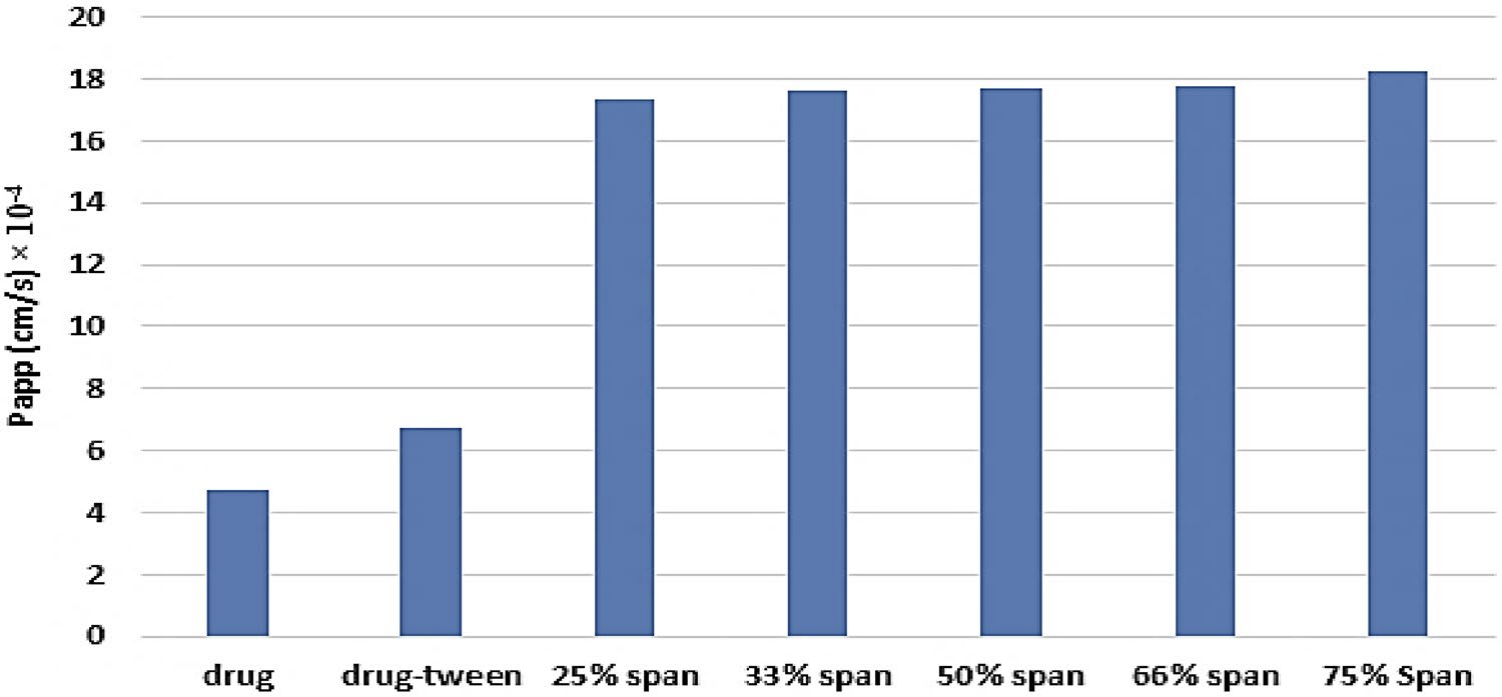
Histogram representation of the apparent permeability of metformin (M-to-S) from the pure drug, drug-tween, and drug-sorbitan monostearate solid dispersion products.

Metformin HCl is a class III drug (highly water-soluble). Entrapment of the drug in sorbitan monostearate led to a huge increasing its partition coefficient indicating the entrapment of the drug occurred in the polar part of the surfactant. This led to the change of the drug from hydrophilic to the lipophilic entrapped image in sorbitan monostearate. The formed image led to the previously reported increasing the partition coefficient and decreasing the drug release. Emulsification of the image by tween 80, led to a marked increasing the apparent permeability and consequently the total permeability of the drug. Emulsification of the image by tween 80 may be changed the image surface from lipophilic to hydrophilic and that may explain the absence of the lag time and the presence of the intercept with y abscissa in concentration. Changing the formed image surface to hydrophilic by adding tween 80 may be led to increasing the Paracellular pathway of the image-encapsulated drug. The similarity of the intercept values to the drug-tween indicating the same permeation mechanism of the image to the drug itself (paracellular pathway). These results could be also confirmed from the histogram representation of the total drug permeation Fig. 4.

The similarity of the drug permeation profiles, the permeability coefficient, total permeation percent, and drug absorption enhancement percent from its different solid dispersion products prepared with different ratios suggesting that the role of sorbitan monostearate is only entrapment of the drug in its polar part as an image. This image entrapment process represents the drug permeation driving force, which is responsible for the paracellular permeation enhancement effect and this effect does not affect all drug-matrix ratios.

### Pharmacodynamics evolution of the metformin HCl products

**Figure 5:**
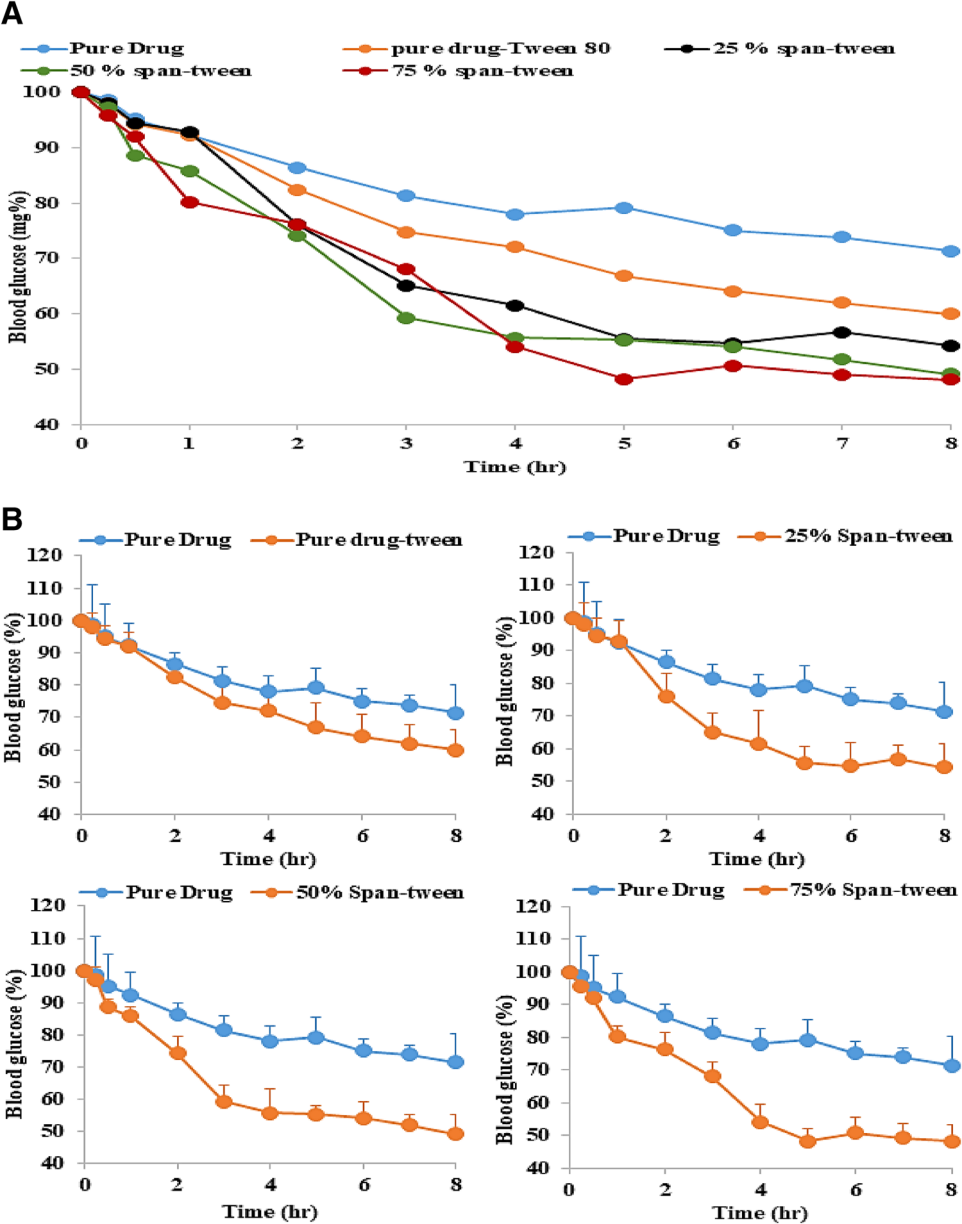
(**A**) Profiles of the change in glucose levels percent versus time. (**B**) Profiles of the change in glucose levels percent versus time (SD error bar).

Evaluation of the in-vivo effect of metformin hydrochloride is monitored by using its pharmacodynamics marker parameter (lowering the blood glucose level after oral administration). The blood glucose level was measured before the drug administration, which represents the glucose level at zero time. After the drug administration, the plasma glucose level is monitored as a function of time. The blood glucose level would be expressed as a percent and the profile of drug glucose concentration is plotted as a function of time Fig. 5A,B.

A drop in the blood glucose level could be noticed from the first hour after oral drug administration. The effect of the high-water solubility of the drug and its paracellular pathway could be also reported as a result of the beginning of dropping of the blood glucose level after 15 min. The maximum and rate of blood glucose level from drug-tween are higher than that from drug alone which confirms the results of the drug permeation through the modified permeation non everted sac test. Expected results could be noticed from the glucose dropping level as a result of administration of drug dispersed in sorbitan monostearate matrix. A clear significant difference between the pharmacodynamic effect of the pure drug and the dropping of glucose level after administration of lower, middle, or higher drug entrapped in sorbitan monostearate at p ˂ 0.05 on applying ANOVA test was found.

From Fig. 5A, it can be noticed that the dropping of glucose level profile after administration of lower (25%), middle (50%), and higher (75%) drug entrapped in sorbitan monostearate are intersecting at more than one point to the degree of congruence. Applying the ANOA test concluded there is no significant difference between the pharmacodynamic effect (dropping of glucose level) of the three products at p ˂ 0.05, which confirms again the drug permeation results from the modified non-everted sac technique. It should be reported that each point represents the mean of 6 blood glucose level measurements at that time with standard deviation as shown in Fig. 5B.

The area above the curves (AAC) was calculated from Fig. 5. The results are tabulated in Table 4. Drug pharmacodynamic enhancement percent was calculated according to Eq. (10).

**Table 4:** The Data of the reduction of blood glucose level and the area above the blood glucose level versus time curve, obtained after oral administration of different Metformin HCl products to diabetic rats.

Reduction of blood glucose (mg/dl)					
Time (h)	Drug	Drug/tween	25%	50%	75%
0.0	0.0	0.0	0.0	0.0	0.0
0.25	9.75 (± 57.8)	10.75 (± 23.0)	10.6 (± 33.7)	15.0 (± 20.1)	22.0 (± 6.5)
0.5	16.75 (± 42.1)	18.25 (± 5.7)	18.6 (± 60.9)	44.0 (± 28.6)	19.0 (± 9.6)
1.0	13 (± 37.4)	10.25 (± 20.1)	8.3 (± 7.6)	14.3 (± 7.5)	59.3 (± 5.1)
2.0	28.25 (± 20.0)	49.25 (± 35.0)	86.6 (± 66.4)	59.3 (± 27.4)	20.0 (± 10.5)
3.0	25.25 (± 12.5)	38.25 (± 14.2)	56.6 (± 9.5)	76.0 (± 6.6)	41.7 (± 8.1)
4.0	16.25 (± 9.0)	13 (± 21.1)	20.3 (± 49.0)	19.0 (± 24.1)	70.3 (± 9.1)
5.0	− 5.3 (± 23.1)	26.5 (± 46.2)	28.3 (± 72.6)	1.3 (± 37.2)	30.0 (± 15.4)
6.0	19.5 (± 16.0)	13.25 (± 33.0)	5 (± 37.0)	6.3 (± 17.2)	− 12.7 (± 9.9)
7.0	6 (± 6.4)	10.5 (± 17.7)	− 11.0 (± 35.5)	11.7 (± 12.3)	8.3 (± 4.0)
8.0	13.75 (± 31.1)	10 (± 16.2)	14.6 (± 55.1)	14.0 (± 25.0)	4.3 (± 7.1)
AAC (mg h/dl)	882.63 (± 231.4)	1067.8 (± 115.9)	1355.8 (± 164.2)	1473.7 ± 185.2)	1512.0 (± 141.9)
Pharmacodynamic enhancement %		20.98	53.61	66.97	71.31
Mean pharmacodynamic enhancement %				63.96 (± 9.23)	

Values between brackets are S.E.M. (n = 6).

10 $$\mathrm{Drug\, pharmacodynamic \,enhancement \,\% }=\frac{(\mathrm{AAC \,of \,treated \,drug}-\mathrm{AAC \,of \,pure \,drug})}{(\mathrm{AAC \,of \,pure \,drug})}\times 100$$

From Table 4, it can be noticed that the drug pharmacodynamic enhancement % increased according to the following order: 75% ˃ 50% ˃ 25% ˃ drug-tween. The mean of pharmacodynamic enhancement percent is 64%, which is equal to drug absorption enhancement effect % as a result of drug molecular dispersion in sorbitan monostearate Table 3.

### Drug intestinal permeation—pharmacodynamic correlation

FDA defined in-vitro-in-vivo correlation (IVIVC) as “a predictive mathematical model describing the relationship between an in vitro property of a dosage form and a relevant in vivo response”. In general, the in vitro property is the rate or extent of drug dissolution or release while the in vivo response is the plasma drug concentration or amount of drug absorbed^39–42^. FDA guidance described 4 levels for IVIVC which are A, B, C, and multiple C^40^. In this study, level A was selected because of its highest category of correlation since it correlates a point-to-point relationship^41,42^.

**Figure 6:**
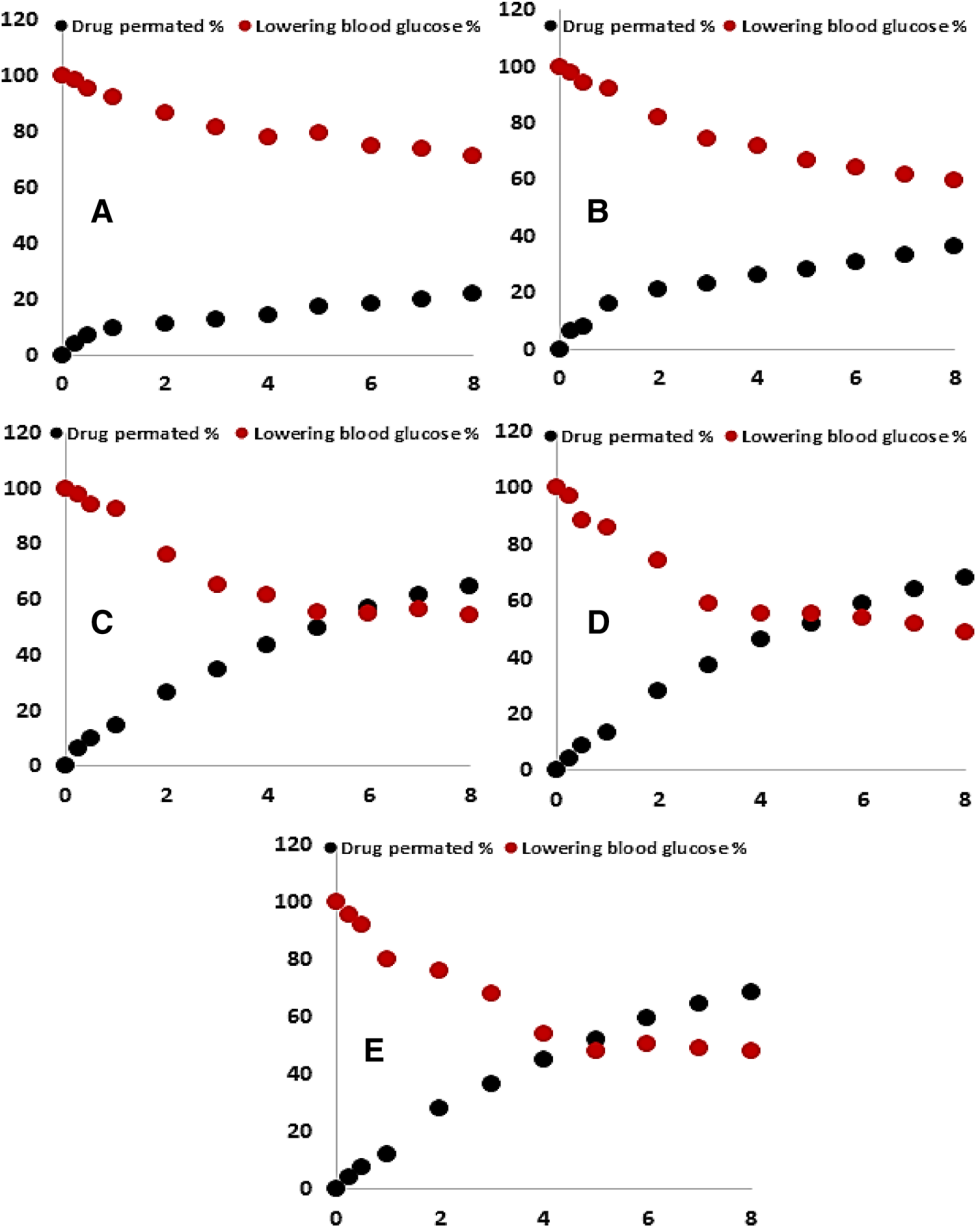
Point to point (level A) correlation of the drug permeation profiles and its pharmacodynamics effect: (**A**) metformin HCL; (**B**) metformin-tween 80; (**C**) 25% drug in span-tween. (**D**) 50% drug in span-tween; (**E**) 75% drug in span-tween.

The pharmacodynamic marker of metformin is its dropping effect on the blood glucose level. Therefore, it was tried to correlate (point-to-point correlation) the percentage of drug permeated and its dropping of blood glucose level percentage. From Fig. 6A–E, it can be noticed that the drug permeation percent curve is the opposite superimposed to the blood glucose dropping percent curve. That is maybe due to the glucose dropping effect is dependent on the blood drug concentration. The area between the two curves would be decreased by increasing the percent of drug molecular dispersed in the span matrix until intercepted at the special point.

Since Level A correlation is a linear relationship between two variables, it was tried to create a mathematic line correlation between the drug permeation enhancing percent and its pharmacodynamics enhancing percent (dropping of glucose level) as a point-to-point correlation. Table 5 shows the value of the correlation coefficient in every case is high enough to conclude a linear correlation between the drug permeation enhancing percent and its pharmacodynamic enhancing percent. Decreasing the slope values of the products than from both drug alone and drug plus tween confirm the drug penetration enhancements, which is consequently lead to more dropping of blood glucose level. The nearly similar slope values of the low, middle, and high drug entrapment products are in agreement with the insignificant difference in the drug permeability and pharmacodynamic effect between the three products. In addition, the results supported the conclusion about the drug permeation driving force is its molecular dispersion in the sorbitan monostearate suggesting image, which is not dependent on the percentage of drug entrapped. Decreasing the intercept values of the products from the pure drug may be, indicates the unstatutable paracellular pathway of the drug absorption from the suggested image, which facing the pure drug absorption.

**Table 5:** Correlation data of drug permeation and pharmacodynamics profile:

	r^2^	Slope	Intercept
Metformin HCl	0.948	− 1.471	102.89
Metformin plus tween 80	0.950	− 1.237	104.73
25% metformin SD	0.944	− 0.791	100.11
50% metformin SD	0.951	− 0.755	96.231
75% metformin SD	0.949	− 0.783	96.162

**Figure 7:**
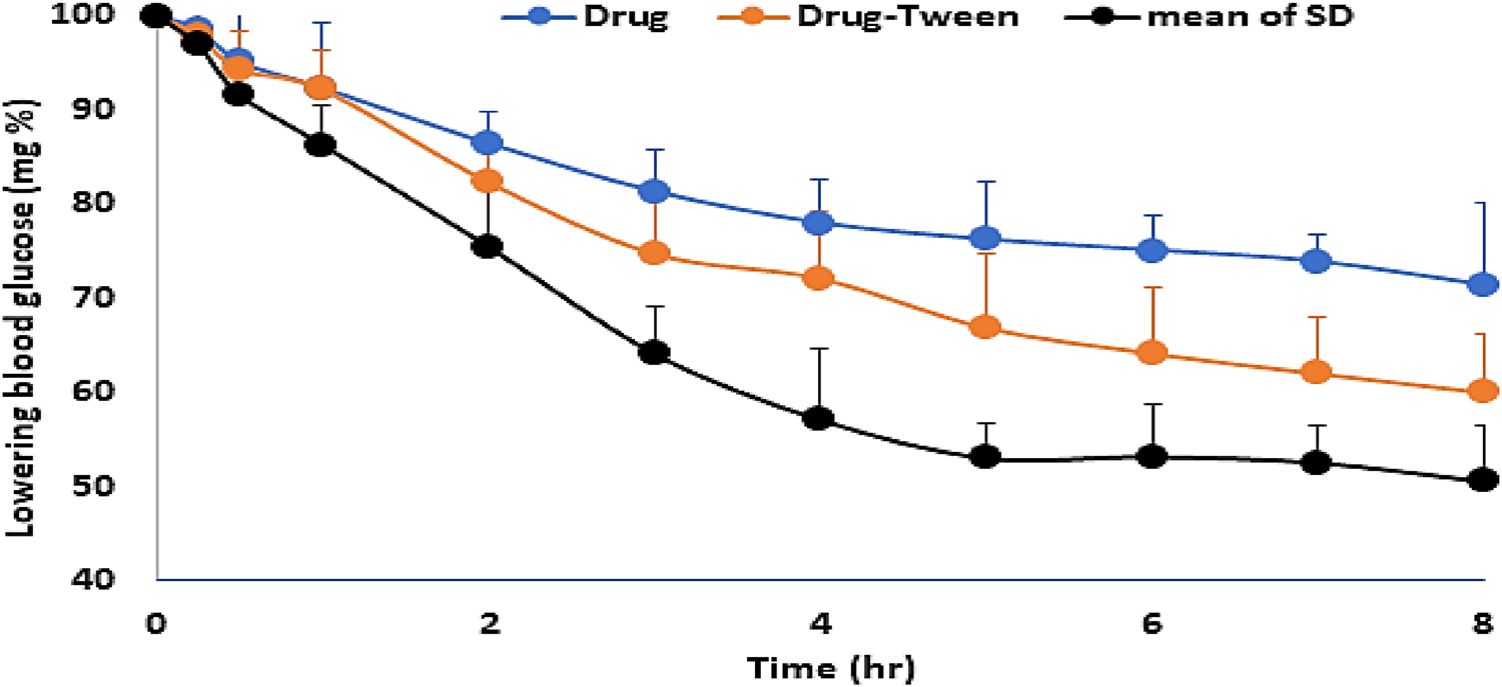
Profiles of the change in glucose levels percent versus time (SD error bar).

From the above study, it was found the following: **1.** the drug permeation profiles from all drug molecular dispersed products in sorbitan monostearate (low, middle and high) are found to be overlapped to each other. **2.** The drug pharmacodynamic effect of the prepared products is intersecting at more than one point to the degree of congruence. **3.** Applying the ANOA test showed there is no significant difference between the pharmacodynamic effect (dropping of glucose level) of the three-drug molecular dispersed products at p ˂ 0.05. **4.** The mean drug absorption (permeation) percent is equal to the mean pharmacodynamic percent. Accordingly, it can be reported that the paracellular enhancement of sorbitan monostearate to metformin is based on its dispersion in the matrix, which is confirmed by the author before^7^, and the enhancement does not depend on the drug–matrix ratio. This conclusion is supported by the results of the ANOVA test, which shows no significant difference between the drug products' pharmacodynamic effects. Accordingly, the mean pharmacodynamic effect of the low, middle and high drug molecular dispersed in the matrix was calculated and is represented in Fig. 7 with bar standard deviation.

## Conclusion

From this study, it can be concluded that the permeability problem facing metformin, as an example for the class III drugs according to BSC classification, may be solved by its dispersion in sorbitan monostearate. Sorbitan monostearate is widely used in food industries and its uses in pharmaceutical technology would be harmless. The high hydrophilicity property of the Class III drug solubilizes them in the polar part of sorbitan monostearate in a special image, which may be theoretically micelle form. The image suggested by the authors (micelle) represents the penetration driving force of class III group drugs via the paracellular pathway. This image, which needs more investigation, does not depend on the drug-matrix ratio. Permeability enhancement could also test by using the author's suggested modified non-everted sac technique. This conclusion is based on an excellent relationship found between the drug permeation percent and its pharmacodynamic effect percent. Improving drug permeability leads to improving the drug bioavailability and consequently leads to decreasing the drug dose and side effects to the patient. At the same time decreasing the cost for the pharmaceutical industry as a result of decreasing the raw materials required, in addition to the simplicity and reproducibility of the technique used, encourage to suggest the technique for its dual effects.

## Data Availability

The raw data supporting the conclusions of this manuscript will be made available by the authors, without undue reservation, to any qualified researcher.
